# Editorial: Benefits and risks of drug combination therapy for chronic metabolic diseases

**DOI:** 10.3389/fendo.2024.1390248

**Published:** 2024-03-07

**Authors:** Tingting Qiu, Dan Yan

**Affiliations:** Beijing Institute of Clinical Pharmacy, Beijing Friendship Hospital, Capital Medical University, Beijing, China

**Keywords:** metabolic disease, combination therapy, gut microbiome, traditional Chinese, network pharmacology

Chronic metabolic diseases are becoming increasingly prevalent and have become major public health issues worldwide. The primary metabolic diseases encompassed obesity, diabetes, non-alcoholic fatty liver disease, hyperlipidemia, and gout. Given the multifactorial nature of metabolism-related diseases, their prevention and treatment necessities multi-target strategies to tackle the physiological and metabolic impairments.

The current strategies for the management of chronic metabolic diseases included lifestyle modification, balanced diet, appropriate exercise, and pharmacological interventions. However, due to the multifaceted pathophysiology underlying these metabolic disorders, developing effective therapeutic and preventive measures has so far been challenging. Current therapeutics for metabolic diseases varied in their ability to improve patient outcomes. For instance, antidiabetic drugs may elicit varying response in patients, some of which may be less favorable or even lead to adverse outcomes. To overcome these challenges, combining medications with diverse mechanism of actions held great promises to produce additive or synergistic effects aiming at reducing macrovascular and microvascular complications associated with metabolic diseases. Xie et al. reviewed that combinations therapy offers significant advantages for diabetes management. Firstly, combination therapy could regulate diabetes through multiple mechanisms and pathways, which may compensate for the limitations of monotherapy and achieve better glucose control. Secondly, combination therapy can potentially allow for the dosage reduction of individual therapies, thereby alleviating the adverse events and improving the patient tolerance. Finally, combination therapy could be tailored to specific patient conditions to provide more personalized treatment strategies.

Lifestyle modification, especially dietary habits, remains the fundamental strategy for the management of metabolic-related diseases. The mendelian randomization analysis conducted by Yu et al. revealed that tea intake has no significant genetic predisposition effect on uric acid, gout, or idiopathic gout. However, they observed that tea intake does have a significant effect on the genetic predisposition of gout due to impairment of renal function. This effect may be attributed to its role in protecting renal function and regulating intestinal function. Therefore, tea intake could be encouraged as a potential dietary treatment for patients with gout associated with impairment of renal function. Chen et al. preformed a systematic review and meta-analysis to investigate the effect of supplementation with probiotics or synbiotics on cardiovascular risk factors in patients with metabolic syndrome. This analysis suggested that compared with the control group, supplementation with probiotics or synbiotics was significantly more effective in decreasing body mass index, low-density lipoprotein and fasting blood glucose, while no additional advantage was observed in the improvement of systolic blood pressure.

Regarding the new treatment modality for diabetes complications, OuYang et al. summarized and analyzed the effectiveness of platelet-rich plasma (PRP) in the treatment of diabetic foot ulcer (DFU). The meta-analysis showed that PRP significantly enhanced the healing rate, and accelerated the healing time when compared to the conventional treatment. However, there was no significant difference observed in reducing the of ulcer area. The mechanism underlying effect of PRP on wound healing primarily involves the release of various bioactive molecules stored in platelets, including PDGF, TGF-β, VEGF, EGF, and adhesion molecules such as fibrin, fibronectin, and hyalenin.

Traditional Chinese medicines (TCMs) possess a unique advantage in the treatment of metabolic disease owning to its ability to regulate multiple targets simultaneously. Hu et al. employ a bioinformatics driven approach in conjunction with network pharmacology to comprehensively explore the therapeutic mechanism of Xuebifang (XBF) in the context of diabetic peripheral neuropathy (DPN). The analysis demonstrated the crucial targets modulated by XBF in treating DPN encompass TNF, IL6, IL1B, MMP9, and PPARG. These findings were further supported by immune infiltration analysis and localization of immune organs and cells. Yang et al. systematically explored the possible therapeutical targets and biological signal pathways of Chaihu Anxin capsule against depression using network pharmacology and molecular docking. The most enriched pathways identified were the AGE-RAGE signal pathway in diabetic complications and lipids and atherosclerosis. Subsequent molecular docking indicated direct interactions between quercetin, beta-sitosterol and kaempferol, and IL6, IL1B, AKT1, TP53, and STAT3. Cao et al. investigated whether Chaihu-Longgu-Muli decoction (CLMD) could modulate CKD-related insomnia in adenine diet-induced CKD mice. The results showed that the administration of CLMD increased nocturnal activity time, the number of activities and sleep time, while sleep latency was decreased. Therefore, this study implied that CLMD can ameliorate sleep disturbances in adenine-induced CKD. The mechanism may be associated with the up-regulation of orexin-A and elevation of phosphorylation level of CaMKK2/AMPK, which further inhibit NF-κB downstream signaling pathways without affecting renal function.

Accumulating evidence suggested that gut microbiome and its metabolites played a crucial role in the onset of numerous metabolic diseases. This implies that probiotic supplements in combined with conventional therapies may represent promising strategy for the management of metabolic diseases. However, the intricate mechanisms underlying the interactions between the gut microbiome and host necessities further investigation. The advent of the technological revolution in artificial intelligence assisted with synthetic biology approaches will play a vital role in the modulating the therapeutic and nutritive potential of probiotics. Likewise, while TCMs have long been widely used for various metabolism diseases, the precise mechanism of action for TCMs for metabolism diseases remain incompletely understood. Leveraging the modern research techniques like epigenomics and bioinformatics technology, researchers have the opportunity to address the current limitations by exploring the chemical underpinnings of the therapeutic effects and potential complications of TCM interventions. There is a growing trend towards employing network pharmacology methods to explore the mechanism of pharmacodynamic substances of TCM prescriptions. Metabolomics has also shown great potential for unravelling the therapeutic mechanisms of TCM, and also in enhancing our understanding of metabolic pathways and disease-associated biomarkers more broadly.

The primary goal of combination treatment for metabolism diseases is to optimize the likelihood of attaining the desired therapeutical goals, while minimizing unnecessary side effects. It is envisioned that in the future, the combination therapy regime should be individualized according to the biological features of specific patient subgroups, thereby expediting the translation into the realm of “precision medicine”.

## Author contributions

TQ: Writing – original draft. DY: Writing – review & editing, Validation, Supervision, Funding acquisition, Conceptualization.

